# A systematic review of emerging RNA markers in thyroid fine needle aspiration cytology samples: advancements and challenges

**DOI:** 10.1007/s12020-025-04266-z

**Published:** 2025-05-09

**Authors:** Gamze Sönmez, Uğur Ünlütürk

**Affiliations:** 1 Department of Medical Biochemistry, Hacettepe University School of Medicine, Ankara, Turkey; 2 Division of Endocrinology and Metabolism, Department of Internal Medicine, Hacettepe University School of Medicine, Ankara, Turkey

**Keywords:** Thyroid cancer, Molecular marker, Diagnosis, Fine-needle aspiration, miRNAs, lncRNAs

## Abstract

**Background:**

Significant advances have been made in detecting RNA markers that may indicate malignancy in fine needle aspiration cytology (FNAC) samples.

**Objective:**

To review the roles of protein-coding and non-coding RNAs in differentiating between malignant and benign thyroid nodules.

**Methods:**

A comprehensive literature search using PubMed, Science Direct, Web of Science, and SCOPUS databases was performed. We searched up until September 2024 and complemented by manual citation search.

**Results:**

A total of 28 full-text articles were reviewed, encompassing 5770 FNAC samples, which included 3489 benign lesions and 2281 malignant lesions. The studies identified 43 messenger RNAs (mRNAs), 16 microRNAs (miRNAs), and 3 long non-coding RNAs (lncRNAs) that have the potential to distinguish malignant nodules. Among the mRNAs, *PAPPA*, *TIMP1*, and *HMGA2*, as well as the miRNAs, miR-146b, miR-375 and miR-222, appear to be the most promising molecules for diagnosis.

**Conclusion:**

Numerous RNA markers have been shown to differentiate malignant from benign lesions. However, there is still a lack of patient-specific classification for thyroid cancer subtypes. Additionally, future studies should prioritize using a combination of molecular markers rather than relying on individual ones. Although current research mainly focuses on identifying cancer-specific molecules, it is important for future studies to shift towards a more patient-specific approach.

## Introduction

The prevalence of thyroid nodules has increased over the last few decades due to the widespread use of imaging techniques [1]. Any thyroid nodule detection requires further assessment due to the potential for indicating thyroid malignancy. The ultrasound-guided fine needle aspiration cytology (FNAC) procedure is used as a preoperative diagnostic method for evaluating thyroid nodules [2]. The Bethesda System for Reporting Thyroid Cytopathology (TBSRTC) atlas has emerged as a critical tool for clinicians and researchers involved in thyroid disease management. It offers a standardized approach for reporting thyroid cytopathology, consisting of six categories to enhance the precision and consistency of thyroid nodule diagnoses. Each category includes an estimated risk of thyroid cancer and recommended guidelines.

However, managing lesions categorized under the third category in TBSRTC, termed atypia of undetermined significance (AUS), poses significant challenges. While studies demonstrate variability in the malignancy rate within AUS, TBSRTC indicates a malignancy rate ranging between 20 and 32% for AUS [3, 4]. There is a growing need for molecular markers to assist in diagnosing nodules classified as AUS. In the last decade, significant developments have been made in developing molecular markers that can be used to detect malignancy in FNAC samples. These markers include gene mutation panels and gene expression classifiers [5]. Validation studies have demonstrated that commercially available molecular tests exhibit high sensitivity and negative predictive values in diagnosing malignancy, although some limitations in specificity have been observed. In recent years, studies evaluating RNA markers in diagnosing malignancy in FNAC samples have been increasing; however, there is yet no comprehensive review in the literature on this issue.

This review aims to explore the roles of protein-coding and non-coding RNAs, particularly microRNAs (miRNAs) and long non-coding RNAs (lncRNAs), as potential markers for malignancy in thyroid FNAC samples. Specifically, we examine their diagnostic accuracy to enhance thyroid cancer diagnosis.

## Materials and methods

This systematic review followed The Preferred Reporting Items in Systematic Reviews and Meta-Analysis guidelines.

### Literature search

**Fig. 1:**
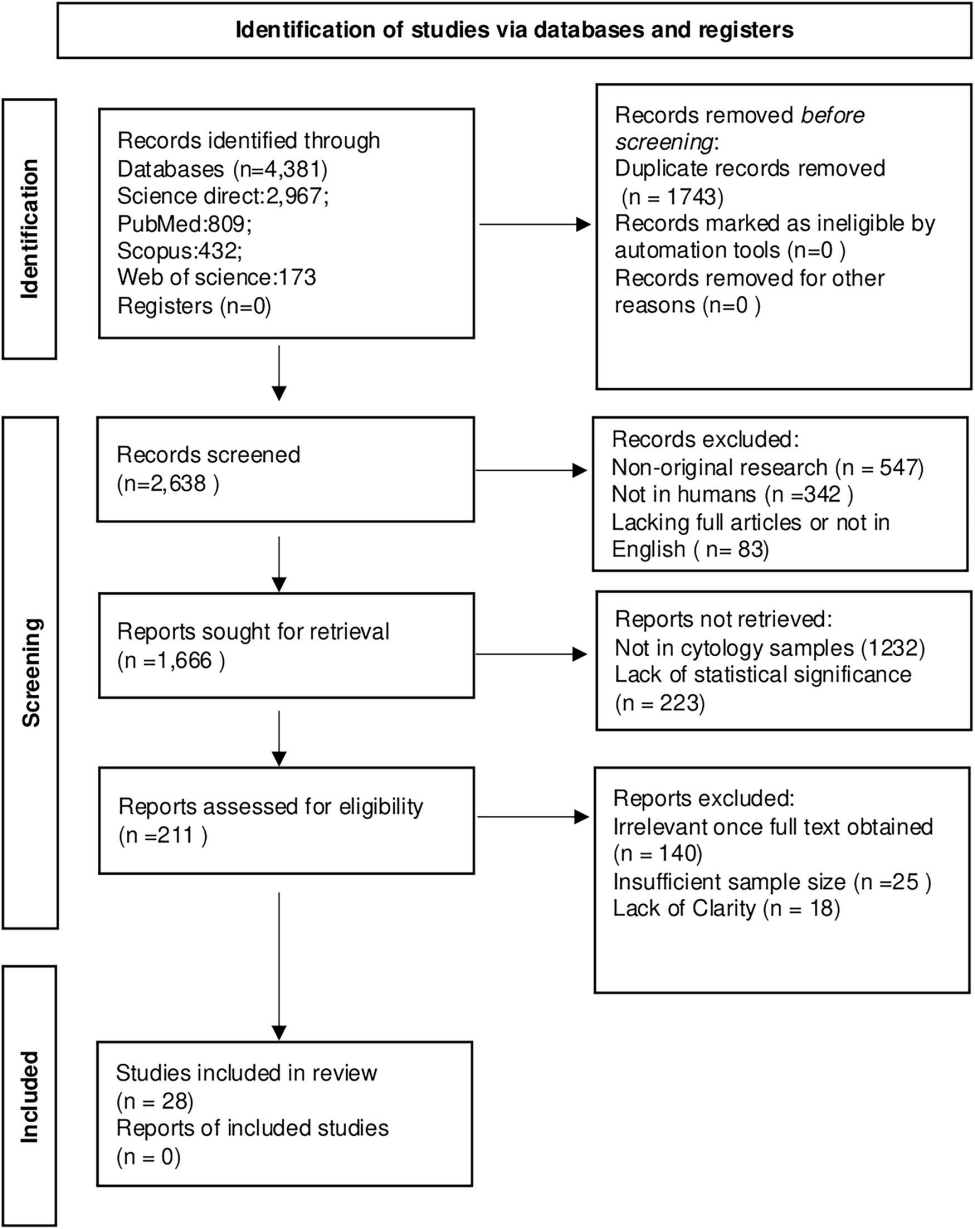
PRISMA 2020 flow diagram for systematic review

A comprehensive literature search was performed using PubMed, Web of Science, Science Direct, and SCOPUS databases. The following search terms were used in all databases: (“thyroid cancer” OR “thyroid nodule” OR “thyroid”) AND (“fine needle aspiration” OR “fine needle aspiration cytology” OR “fine needle aspirates”) AND (“molecular marker” OR “biomarker” OR “RNA” OR “miRNA” OR “lncRNA”). The electronic databases were last utilized for searches on September 23, 2024. Only original research articles published in English from January 1998 to August 2024 were considered. The search results from each database were exported into ProQuest RefWorks for the purpose of removing duplicate entries and conducting further review. The findings of this systematic review were presented in adherence to the PRISMA (Preferred Reporting Items for Systematic Reviews and Meta- Analyses) guidelines (available at http://www.prisma-statement.org) (Fig. 1). We have registered the protocol in the International Prospective Register of Systematic Reviews database (PROSPERO) at https://www.crd.york.ac.uk/PROSPERO, with the number CRD42024593405.

### Study selection and eligibility criteria

Inclusion criteria encompass (i) prospective or retrospective published studies with ethical clearance, (ii) alignment of research content with the topic of interest, (iii) inclusion of comparative studies between benign and malign nodules, (iv) studies comparing benign and malignant nodules with statistically significant results (*p* < 0.05), (v). histopathological confirmation of thyroid malignancies and (vi) all types of thyroid malignancies were considered. Exclusion criteria encompass (i) non-original articles (reviews, case reports), (ii) studies with sample sizes less than ten for benign or malignant lesions (iii) studies involving animal models (e.g., mouse and rat) or unrelated to the subject matter, (iv) in instances where the same study was published multiple times, (v) publications in languages other than English and (vi) significant amounts of missing or inadequate data.

### Data extraction

Two reviewers extracted study characteristics and results from the included studies, including details such as the first author’s name, publication year, country or region, study design, sample size, sex distribution, age range, number of tumor recurrence cases, and complications. Data extraction was conducted independently by two reviewers (GS and UÜ).

### Study quality assessment

The methodological quality of the included studies was assessed using the Quality Assessment of Diagnostic Accuracy Studies-2 (QUADAS-2) tool. This tool evaluates studies based on four criteria: “patient selection,” “index test,” “reference standard,” and “flow and timing” [6]. Each criterion was examined for risk of bias and applicability. Responses were categorized as “yes,” “no,” or “unclear,” with “yes” indicating a low risk of bias and “no” or “unclear” indicating a higher risk or unclear risk.

## Results

### Search results

The database searches provided a total of 4381 results (Science direct: 2967; PubMed: 809; Scopus: 432 and Web of Science:173). A total of 28 articles published between 1998 and 2024 fulfilled the inclusion criteria and underwent analysis for this research. The extracted data from these studies are summarized in Tables 1, 2 and 3. Among the studies, a total of 5770 fine needle aspiration biopsies were included in our analysis, consisting of 3489 benign and 2281 malignant lesions.

**Table 1:** Studies comparing the expression level of protein-coding RNAs in cases and controls

Marker	Reference	Method	Benign Cases	Malignant Cases	Types of Malignancies	Sensitivity (%)	Spesificity (%)
CCND2	[11]	qRT-PCR	65	65	PTC	67.7	89.2
PAPPA	[15]	qRT-PCR	57	23	PTC	80	82
TIMP1	[17]	qRT-PCR	20	20	PTC	95	85
	[18]	qRT-PCR	22	25	PTC, NIFTP	83	100
	[19]	qRT-PCR	239	132	PTC, ATC, OCC, FTC	100	77
	[20]	MA	11	11	PTC	100	95.4
CHI3L1	[17]	qRT-PCR	20	20	PTC	70	60
HMGA2	[37]	qRT-PCR	192	39	PTC, FTC	91.9	84.5
	[38]	qRT-PCR	210	289	PTC, FTC, OCC, MTC	72	91
	[32]	qRT-PCR	67	28	PTC	80	100
	[20]	MA	11	11	PTC	100	95.4
c-MET	[18]	qRT-PCR	22	25	PTC, FTC, NIFTP	—	—
ARNTL	[18]	qRT-PCR	22	25	PTC, NIFTP	—	—
TROP-2	[29]	qRT-PCR	10	10	PTC	96	87.5
	[20]	MA	11	11	PTC	100	95.4
SLP-2	[29]	qRT-PCR	10	10	PTC	83.3	79.3
CTNNB1	[41]	qRT-PCR	45	29	PTC	—	—
CDH1	[41]	qRT-PCR	45	29	PTC	—	—
CD56	[29]	qRT-PCR	10	10	PTC	86.7	93.1
LGALS3	[20]	MA	11	11	PTC	100	95.4
	[36]	qRT-PCR	93	18	PTC, FTC	78	84
TFF3	[36]	qRT-PCR	93	18	PTC, FTC	78	84
	[20]	MA	11	11	PTC	100	95.4
HGD1	[36]	qRT-PCR	93	18	PTC, FTC	78	84
	[20]	MA	11	11	PTC	100	95.4
ADM3	[36]	qRT-PCR	93	18	PTC, FTC	78	84
ANGPT2	[19]	qRT-PCR	239	132	PTC, FTC, ATC, OCC	100	77
EGFR	[19]	qRT-PCR	239	132	PTC, FTC, ATC, OCC	100	77
EFNB2	[19]	qRT-PCR	239	132	PTC, FTC, ATC, OCC	100	77
TMPRSS4	[19]	qRT-PCR	239	132	PTC, FTC, ATC, OCC	100	77
CK19	[20]	MA	11	11	PTC	100	95.4
UBE2C	[13]	qRT-PCR	54	30	PTC, FTC	100	89
TPO	[20]	MA	11	11	PTC	100	95.4
PTMA	[48]	Semi-qRT-PCR	24	13	PTC, FTC	—	—
PTMS	[48]	Semi-qRT-PCR	24	13	PTC, FTC	—	—
MRC2	[32]	qRT-PCR	67	28	PTC	71	84
SFN	[32]	qRT-PCR	67	28	PTC	71	84
FCGBP	[20]	MA	11	11	PTC	100	95.4
MATN2	[20]	MA	11	11	PTC	100	95.4
RAP1GA1	[20]	MA	11	11	PTC	100	95.4
TIPARP	[20]	MA	11	11	PTC	100	95.4
QPCT	[20]	MA	11	11	PTC	100	95.4
PSD3	[20]	MA	11	11	PTC	100	95.4
DUSP4	[20]	MA	11	11	PTC	100	95.4
ABCC3	[20]	MA	11	11	PTC	100	95.4
DPPIV	[20]	MA	11	11	PTC	100	95.4
PROS1	[20]	MA	11	11	PTC	100	95.4
AAT	[20]	MA	11	11	PTC	100	95.4
FN1	[20]	MA	11	11	PTC	100	95.4
CITED1	[20]	MA	11	11	PTC	100	95.4
LRP4	[20]	MA	11	11	PTC	100	95.4
SH2D1A	[20]	MA	11	11	PTC	100	95.4
SERPINA1	[20]	MA	11	11	PTC	100	95.4

*CCND2* cyclin D2, *PAPPA* pregnancy-associated plasma protein A, *TIMP1* tissue inhibitors of metalloproteinase 1, *CHI3L1* chitinase 3-like 1, *HGF* hepatocyte growth factor, *TROP-2* trophoblast antigen 2, *SLP-2* stomatin-like protein 2, *TFF3* trefoil factor 3, *HGD1* homogentisate 1,2-dioxygenase, *ANGPT2* angiopoietin-2, *EFNB2* ephrin B2, *CK19* cytokeratin 19, *TPO* thyroperoxidase, *ProTα* prothymosin α, *HMGA2* the high mobility group protein 2, *MRC2* mannose receptor C type 2, *SFN* stratifin, *FCGBP* Fc fragment of IgG binding protein, *MATN2* matrilin 2, *RAP1GA1* rap1-GTPase activating protein 1, *TIPARP* TCDD-inducible poly-ADP-ribose polymerase, *QPCT* glutaminyl-peptide cyclotransferase, *PSD3* pleckstrin and sec7 domain containing 3, *DUSP4* dual-specificity phosphatase 4, *ABCC3* ATP-binding cassette, sub-family C, *DPPIV* dipeptidyl peptidase-4, *PROS1* tumour-secreted protein S, *AAT* alpha-1 antitrypsin, *CITED1* glutamic acid and aspartic acid-rich C-terminal domain 1, *LRP4* low-density lipoprotein receptor-related protein 4, *FN1* fibronectin 1, *SH2D1A* SH2 domain protein 1A, *SERPINA1* serine (or cysteine) proteinase inhibitor, *PTC* papillary thyroid cancer, *FTC* follicular thyroid Cancer, *MTC* medullary thyroid carcinoma, *OCC* oncocytic cell carcinoma, *ATC* anaplastic thyroid cancer, *MA* microarray analysis

**Table 2:** Studies comparing the expression level of miRNAs in cases and controls

miRNA	Reference	Up/Down Regulated	Targeted Genes	Benign Cases	Malignant Cases	Types of Malignancies	Sensitivity (%)	Spesificity (%)
miR-181b	[54]	up-regulated	CYLD	204	20	PTC, ATC, MTC	83	83
miR-146b	[38]	up-regulated	SMAD4, IRAK1	210	289	PTC, FTC, OCC, MTC	—	—
	[54]			204	20	PTC, ATC, MTC	83	66
	[55]			90	246	PTC, FTC, MTC, ATC	92.1	82.3
	[57]			54	63	PTC, FTC, MTC, ATC, NIFTP	78	93
	[59]			146	12	PTC	90.9^a^	98.5^a^
	[56]			50	22	PTC, FTC, OCC	100^a^	86^a^
	[65]			13	57	PTC, FTC, OCC, NIFTP	89.1	87.5
	[60]			13	38	PTC	87.8	100
miR-885–5p	[62]	down-regulated	CDK2, HTRA2	23	38	FTC	—	—
miR-222	[56]	up-regulated	p27, p57	50	22	PTC, FTC, OCC	100^a^	86^a^
	[59]			146	12	PTC	90.9^a^	98.5^a^
miR-328	[56]	up-regulated	H2AFX	50	22	PTC, FTC, OCC	100^a^	86^a^
miR-15a	[65]	up-regulated	BCL-2	13	57	PTC, FTC, OCC, NIFTP	72.4	80
miR-197	[56]	up-regulated	ACVR1,TSPAN3	50	22	PTC, FTC, OCC	100^a^	86^a^
miR-21	[56]	up-regulated	TIMP3, TGFBR2	50	22	PTC, FTC, OCC	—	—
miR-484	[63]	down-regulated	—	20	24	FTC	89	87
miR-148b-3p	[63]	down-regulated	—	20	24	FTC	89	87
miR-206	[11]	down-regulated	LMX1B	65	65	PTC	92,3	81,5
miR-221	[59]	up-regulated	RECK	146	12	PTC	90.9^a^	98.5^a^
	[38]			210	289	PTC, FTC, OCC, MTC	—	—
miR-375	[58]	up-regulated	ERBB2	210	289	PTC, FTC, OCC, MTC	100^b^	100^b^
miR-30a-5p	[61]	up-regulated	E2F7, SEPT7	40	44	PTC, OCC	—	—
miR-7	[66]	down-regulated	CKS2	67	28	PTC	100	29
miR-138	[67]	up-regulated	hTERT, THRB	78	47	PTC, OCC	—	—

*CYLD* cylindromatosis, *SMAD4* SMAD family member 4, *IRAK-1* interleukin-1 receptor-associated kinase 1, *CDK2* cyclin-dependent kinase 2, *HTRA* htrA serine peptidase 2, *H2AFX* H2A histone family member X, *ACVR1* activin A receptor type I, *TSPAN3* tetraspanin 3, *TIMP3* metalloproteinase inhibitor 3, *TGFBR2* transforming growth factor beta receptor 2, *LMX1B* LIM homeobox transcription factor 1-beta, *RECK* reversion-inducing cysteine rich protein with Kazal motifs, *ERBB2* receptor tyrosine-protein kinase erbB-2, *E2F7* E2F transcription factor, *SEPT7* septin-7, *CKS2* CDC28 protein kinase regulatory subunit 2, *hTERT* human telomerase reverse transcriptase, *THRB* thyroid hormone receptor beta, *PTC* papillary thyroid cancer, *FTC* follicular thyroid cancer, *MTC* medullary thyroid carcinoma, *OCC* oncocytic cell carcinoma, *ATC* anaplastic thyroid cancer

^a^Combined with other miRNAs

^b^in MTC samples

**Table 3:** Studies comparing the expression level of LncRNAs in cases and controls

LncRNAs	Reference	Up/Down Regulated	Associated Pathway	Benign Cases	Malignant Cases	Types of Malignancies	Sensitivity (%)	Spesificity (%)
LRRC52-AS1	[73]	up-regulated	Via mesenchymal markers N-cadherin, Vimentin	25	20	PTC	83.3	100
LINC02082	[73]	up-regulated	PI3K-AKT signaling pathway	25	20	PTC	88.9	91.3
UNC5B-AS1	[73]	up-regulated	—	25	20	PTC	88.9	81.3

*PTC* papillary thyroid cancer

### General study characteristics

The included studies revealed the identification of 43 messenger RNA (mRNAs), 16 miRNAs and 3 lncRNAs with the potential to distinguish malignant tumors. All 43 mRNA markers were studied in papillary thyroid cancer (PTC) samples, while 18 (42%) were studied in follicular thyroid cancer (FTC) samples, 9 (21%) in oncocytic cell carcinoma samples, 2 (5%) in medullary thyroid cancer (MTC) samples; and 5 (12%) in anaplastic carcinoma samples and 3 (7%) in non-invasive follicular thyroid neoplasm with papillary-like nuclear features (NIFTP). Out of 16 miRNAs, 13 (81%) miRNAs were studied in PTC samples, 10 (63%) miRNAs in oncocytic cell carcinoma, 10 (63%) miRNAs in FTC samples, 4 (20%) miRNAs in MTC samples, and 3 (19%) miRNA in NIFTP samples. All lncRNAs were studied in PTC samples.

### Study quality assessment

**Fig. 2:**
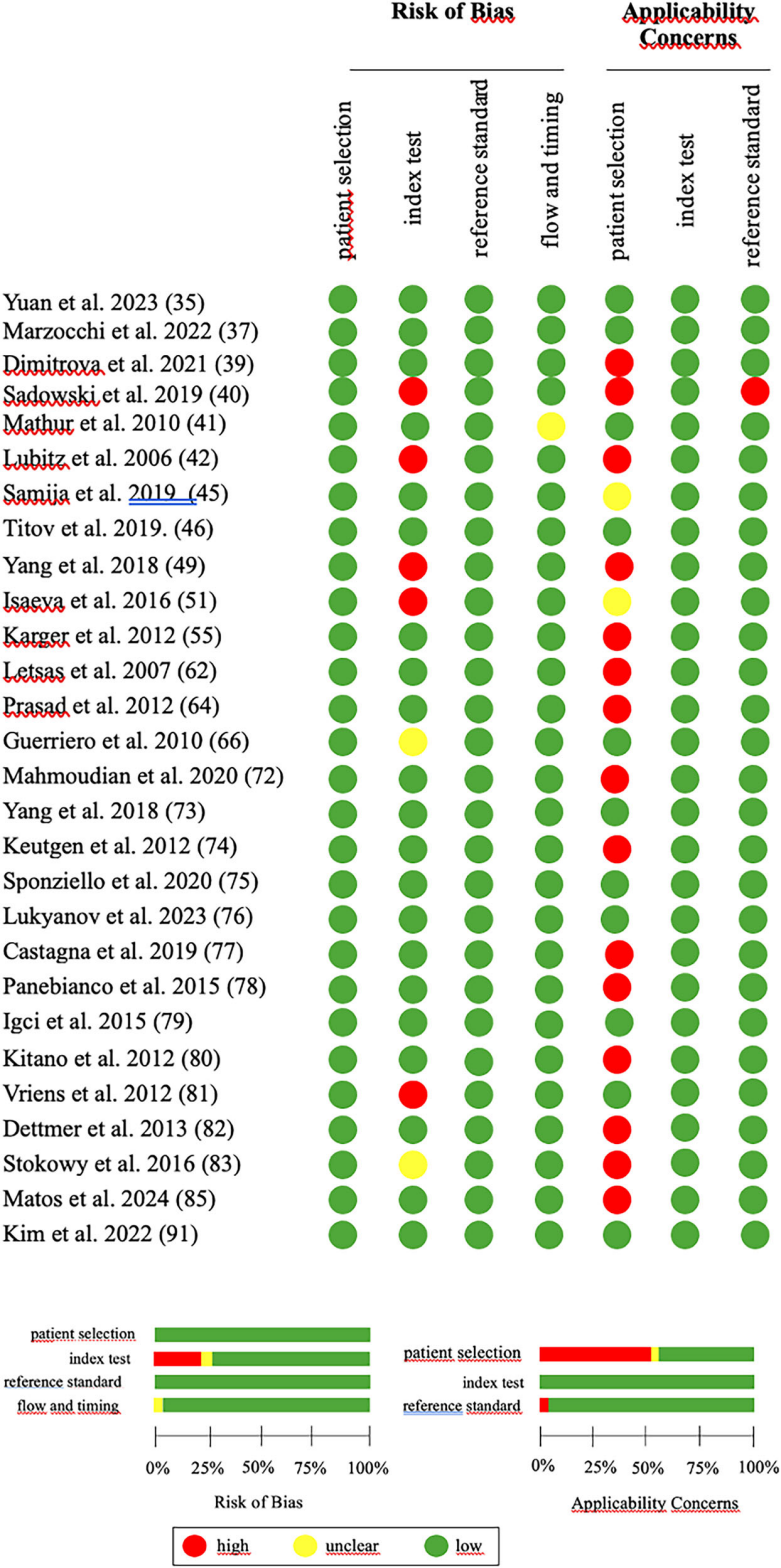
Evaluation of bias risk and relevance issues in the included studies using the QUADAS-2 tool

The methodological quality of the 28 studies was evaluated using the QUADAS-2 tool. Fig. 2 displays a summary of the risk of bias evaluation for the 28 studies included. Overall, the quality of the included studies was deemed satisfactory and eligible. However, the study identified significant concerns related to applicability, particularly concerning the patient selection. These concerns were primarily due to the varying numbers of malignant and benign nodules studied, as well as differences in the types of thyroid cancer examined.

### Coding RNAs as thyroid cancer markers

Coding RNAs, particularly mRNAs, encode proteins that function as enzymes, cellular structures, and signaling molecules [7]. So far, the expression of numerous genes have been demonstrated in FNAC samples using various methods for distinguishing between benign and malignant tissues. Table 1 provides a summary of these molecules.

### Cell cycle and proliferation markers

Cell cycle and proliferation markers play a significant role in understanding thyroid cancer biology, particularly in relation to tumor aggressiveness and progression. Key markers involved in the regulation of the cell cycle in thyroid cancer include cyclins, cyclin-dependent kinases (CDKs), and their inhibitors, which collectively orchestrate the transition between different phases of the cell cycle [8].

#### CCND2

Cyclin D2, encoded by *CCND2*, is a notable member of the cyclin family, characterized by its dynamic changes during the cell cycle [9]. Its increased expression in thyroid cancer tissues, often linked to the transcription factor PITX2, highlights its role in tumorigenesis [10]. Although its sensitivity in diagnosing PTC remains low, combining *CCND2* expression with the miR-206, which targets it, has demonstrated potential [11]. Importantly, miR-206 expression was found to be low, whereas *CCND2* expression was elevated in the FNAC samples of thyroid cancer patients, indicating a negative correlation between *CCND2* and miR-206. Functional studies are needed to clarify the mechanisms underlying the negative correlation between miR-206 and *CCND2*.

#### UBE2C

Similarly, *UBE2C*, encoding UbcH10, a key enzyme in cell cycle regulation, is minimally detectable in normal thyroid cells but significantly overexpressed in thyroid carcinoma cell lines [12]. This marker has shown promise for enhancing malignancy identification in thyroid FNACs and could be incorporated into quantitative RT-PCR (qRT-PCR) assays for more precise diagnostics [13].

### Metalloproteinases and tumor microenvironment

In addition to cell cycle markers, the tumor microenvironment (TME) plays a crucial role in the progression of thyroid cancer. Like other tumors, thyroid cancers possess a unique TME that influences various aspects of tumor behavior. Consequently, molecules involved in the biology of the tumor microenvironment have garnered significant attention.

#### PAPPA

Pregnancy-associated plasma protein-A (*PAPPA*), a metalloproteinase involved in IGF-1 release, has been observed at elevated levels in Bethesda category V–VI nodules, correlating with increased malignancy risk [14]. Furthermore, a statistically significant trend showed increasing *PAPPA* levels from low to high risk of malignancy [15].

#### TIMP1

Tissue inhibitors of metalloproteinase 1 (*TIMP1)* plays a role in initiating and progressing thyroid cancer [16]. Several studies have shown its importance in distinguishing between benign and malignant nodules in PTC [17–20]. Dimitrova Inna et al. recognized *TIMP1* recognized as a crucial marker, demonstrating a sensitivity of 95% and a specificity of 85% [17]. Another study developed a scoring system that incorporates FNAC classification, the presence of an *NRAS* variant, and the expression level of *TIMP1*, achieving an accuracy of 67% in diagnosing indeterminate nodules [19].

### Angiogenesis and invasion

Angiogenesis, a crucial process for tumor growth and progression, is not dictated by a single pathway but arises from a complex interplay of various factors and signal transduction systems [21]. As the tumor microenvironment evolves, processes like angiogenesis and invasion become increasingly vital for tumor development and metastasis, allowing tumors to access essential nutrients and spread throughout the body.

#### CHI3L1

CHI3L1 increases the expression of VEGF and its receptor, promoting angiogenesis by activating the JNK and p38 signaling pathways [22]. It is associated with PTC that has metastasized to lymph nodes [23], and only one study showed *CHI3L1* as a useful marker in FNAC samples for differentiating PTC nodules [17]. However, sensitivity and specificity were low (sensitivity:70% and specificity: 60%). Interestingly, they did not observe a correlation between the mRNA expression of *CHI3L1* and the serum levels of its corresponding protein product. This discrepancy may be attributed to degradation processes and post-translational modifications occurring within the cell.

#### ANGPT2

Angiopoietin-2 (*ANGPT2)* is a ligand for Tie2, a receptor tyrosine kinase predominantly expressed on endothelial cells. It destabilizes blood vessels and plays a key role in regulating their maturation [24]. It has been determined that *ANGPT2* expression can differentiate between benign and malignant thyroid tumors with high sensitivity (100%) but low specificity (77%) [19]. The findings from this study also highlight the potential of other molecular markers alongside *ANGPT2*, including epidermal growth factor receptor (*EGFR*), Ephrin B2 (*EFNB2*), and transmembrane serine protease 4 (*TMPRSS4*), which are involved in crucial processes such as cell migration, vascularization, and invasion [25, 26]. Incorporating these additional markers may improve diagnostic accuracy by providing a comprehensive profile of tumor characteristics, thereby enhancing the ability to distinguish malignant nodules.

#### SLP-2

Stomatin-like protein 2 has been shown to control the production of reactive oxygen species and to enhance the motility and angiogenesis of PTC [27]. Bartolome et al. indicated that the upregulation of *SLP-2* in surgical samples is closely associated with the aggressiveness of PTC and the BRAF p.V600E variant [28]. A small sample study identified *SLP-2* as a differentiating marker in FNAC of PTC nodules. However, the study was unable to establish a correlation between *SLP-2* levels and various clinicopathological factors [29].

#### MRC2

Mannose Receptor C Type 2 (*MRC2*) has been associated with metastasis in different types of cancers [30]. A recent study using The Cancer Genome Atlas database demonstrated that papillary adenocarcinomas of the thyroid exhibited moderate to strong cytoplasmic positivity in the tumor cells [31]. Prasad et al. demonstrated that *MRC2*, along with Stratifin (*SFN*), could assist in distinguishing between benign and malignant thyroid nodules with inconclusive cytology [32]. Utilizing a three-gene model (*HMGA2*, *MRC2*, and *SFN*), they achieved sensitivity of 71% and specificity of 84% for detecting malignant nodules. However, since follicular cancers were not represented in that study, it cannot be concluded that this classifier is capable of distinguishing between follicular adenoma and carcinoma.

#### TFF3

Trefoil factor 3 (*TFF3*) has been shown to inhibit the migration of thyroid cancer cells while promoting apoptosis, suggesting a dual role in both limiting cancer spread and inducing cell death [33]. In addition to *TFF3*, other markers such as homogentisate 1,2-dioxygenase (*HGD1*) and *ADM3*, a member of the adrenomedullin family, are also recognized for their involvement in thyroid cancer biology, albeit with less clarity regarding their specific roles [34, 35]. Karger et al. noted significant variations in the expression levels of these markers, including *LGALS3*, which encodes the Galectin-3 protein, when comparing thyroid cancer tissues to benign nodules and healthy thyroid tissue [36]. Such variations highlight the potential for these markers to enhance our understanding of the invasive characteristics and pathogenesis of thyroid cancer.

#### HMGA2

High-mobility group AT-hook 2 (*HMGA2*) is s a non-histone chromatin-binding protein that is involved in the regulation of gene expression and structural changes in chromatin organization. It plays a critical role in cell growth and metastasis and is recognized as a marker of malignancy in thyroid cancer [37]. A study conducted with a large sample demonstrated that *HMGA2* can effectively differentiate between benign and malignant tumors among follicular neoplasms, with a sensitivity of 72% [38]. This means that while *HMGA2* is a valuable tool in identifying malignancies, there remains a 28% chance of missing positive cases.

### Cell death and adhesion

Programmed cell death (apoptosis), is essential for maintaining physiological homeostasis by eliminating damaged cells. However, in cancer, this process can become dysregulated, leading to inadequate removal of malignant cells or excessive death of normal cells [39]. The regulation of cell adhesion is closely linked to these cell death mechanisms; compromised adhesion can result in abnormal apoptosis, enabling cancer cells to evade cell death and promoting tumor progression. Consequently, molecules associated with these pathways may serve as indicators of thyroid cancer progression.

#### CTNNB1

β-Catenin (*CTNNB1*) is essential for preserving cell-cell adhesion and promotes neoplastic growth in a role that is not fully understood [40]. Its interaction with the cytoplasmic tail of E-cadherin (*CDH1*) facilitates cell adhesion within a complex that sustains cell polarity. Isaeva et al. reported a significant decrease in *CTNNB1* gene expression in patients with PTC compared to those with nodular colloid goiter and follicular adenoma [41]. Similarly, *CDH1* gene expression was low in PTC samples; however, E-cadherin was consistently detected on the cell membrane. the findings indicate that while the gene expression levels of both *CTNNB1* and *CDH1* are reduced in PTC, E-cadherin protein remains present on the cell membrane. This indicates a potential disruption in the E-cadherin/β-catenin complex that may contribute to altered cell adhesion properties.

#### c-Met

The *MET* gene encodes the c-MET receptor, which is a tyrosine kinase crucial for mediating cellular responses to hepatocyte growth factor. This receptor is integral to processes such as cell proliferation, migration, and survival, making it an attractive target for preoperative cytological diagnostics in thyroid cancer. Its expression is present in approximately 70–90% of PTC samples, while it remains undetectable in normal thyroid tissue [42]. This makes c-MET an attractive target for preoperative cytological diagnostics in thyroid cancer. Cabozantinib, a tyrosine kinase inhibitor targeting c-MET, has demonstrated efficacy in patients with MTC [43]. Therefore, assessing the expression levels of c-MET in FNAC samples of MTC patients may be a valuable approach.

#### TROP-2

Recently, TROP-2, a membrane glycoprotein, has garnered significant attention as a prognostic marker and has been investigated as a promising target for immunotherapy in the treatment of human cancers [44, 45]. Elevated *TROP-2* expression has been noted in thyroid cancers, though some studies report sensitivity as high as 100%, raising concerns about the reliability of these results due to small sample sizes [20, 29].

#### PTMA and PTMS

Prothymosin α (*PTMA*) is a nuclear protein contributing to oncogenesis by stimulating cell growth and inhibiting apoptosis [46]. Parathymosin (*PTMS*) also appears to play a role in cell proliferation [47]. Importantly, *PTMA* and *PTMS* mRNA levels are higher in well-differentiated carcinomas compared to adenomas and goiters, suggesting a correlation with the proliferative activity of thyroid follicular cells [48].

### Other markers

In a pioneering study, Lubitz et al. utilized microarray analysis to evaluate the expression of 25 genes in FNAC samples, marking a significant advance in the literature [20]. They identified several genes with altered expression. For instance, Matrilin 2 (*MATN2*) and Rap1-GTPase activating protein 1 (*RAP1GA1*) were notably underexpressed in thyroid carcinoma samples. In contrast, genes such as TCDD-inducible poly (ADP-ribose) polymerase (*TIPARP*) and Glutaminyl-peptide cyclotransferase (*QPCT*), exhibited overexpression in thyroid carcinoma. However, a limitation of this study was the absence of follicular carcinomas in the cohort, and the small sample size raises questions regarding the reported sensitivity, particularly the claim of 100% accuracy [20].

### Existing limitations

While coding RNAs present a valuable array of markers, not all exhibit equal potential for clinical application. Molecules like *PAPPA, TIMP1*, and *HMGA2* are particularly noteworthy due to their substantial sample sizes and diagnostic utility, making them promising candidates for enhancing thyroid cancer diagnosis. Although these markers demonstrate strong sensitivity and specificity, their reported efficacy may not be applicable in all clinical scenarios. The occurrence of false negatives and inconsistencies in marker expression can lead to missed diagnoses or misinterpretations, ultimately affecting treatment decisions.

A key advantage of coding RNA markers is their ability to provide functional insights into the tumor’s behavior. While classical genetic tests identify specific mutations, coding RNA markers reveal how changes in gene expression influence vital cellular processes such as proliferation and apoptosis. This functional information not only enhances risk stratification but also aids in tailoring more personalized treatment plans for patients with thyroid cancer.

### Non-coding RNAs as thyroid cancer markers

ncRNAs are a diverse class of RNA molecules that do not encode proteins but play crucial roles in various cellular processes and diverse disease spectra. These molecules have garnered significant attention in recent years due to their involvement in regulating gene expression at multiple levels. ncRNAs can be classified into various types, including miRNAs, lncRNAs, circular RNAs (circRNAs), heterogeneous nuclear RNAs (hnRNAs), PIWI-interacting RNAs (piRNAs), ribosomal RNAs (rRNAs), small nuclear RNAs (snRNAs), small nucleolar RNAs (snoRNAs), and transfer RNAs (tRNAs) [49]. Until now, only miRNAs and lncRNAs have been examined in aspiration cytology samples.

### microRNAs

miRNAs, typically around 22 nucleotides in length, comprise a class of small non-coding RNAs [50]. They are primarily transcribed from DNA sequences into primary miRNAs (pri-miRNAs), which are then processed into precursor miRNAs (pre-miRNAs) and ultimately mature miRNAs. Generally, miRNAs function by binding to the 3′ untranslated region (UTR) of target mRNAs, leading to the suppression of gene expression [50, 51]. Recent studies have indicated that the expression levels of specific miRNAs in thyroid tumor tissues correlate with various clinicopathological features, including tumor size, multifocality, capsular invasion, extrathyroidal extension, as well as lymph node and distant metastases [52, 53]. For these reasons, the role of miRNAs has come into play in distinguishing AUS cases as benign or malignant. A summary of the relevant studies is presented in Table 2.

#### miRNAs as diagnostic biomarkers in thyroid cancer

Among specific miRNAs, miR-146b stands out due to its significant upregulation in PTC, which correlates positively with the presence of malignant thyroid tumors. This miRNA holds promise as a potential marker for diagnosing PTC [38, 54–56]. It has also shown the ability to differentiate non-PTC thyroid cancers, despite some studies being limited by small sample sizes [57]. Sponziello et al. proposed a dual-component molecular test that combines mutation analysis with the miR-146b-5p assay to improve the diagnostic precision of thyroid cytology [57]. This combined approach successfully eliminated all three false-negative results encountered in single-component analysis, underscoring the effectiveness of using combined markers.

miR-375 has shown effectiveness in distinguishing MTC from other malignant and benign thyroid tumors [58]. Its utility as a selective marker for MTC could aid clinicians in differentiating this specific type of thyroid cancer from others, thus guiding treatment decisions.

In their study, Castagna et al. found elevated expression levels of miR-146b, miR-222, and miR-221 in nodules categorized as malignant or suspicious for malignancy compared to benign nodules, with a fold change expression of ≥ 5 [59]. Another study suggested a four-gene model, including miR-146b and miR-222, to identify suspicious nodules in cases where cytological analysis is inconclusive and is wild-type BRAF [60].

Further supporting the role of miRNAs in diagnosis, Igci et al. reported significant elevation of miR-30a-5p in serum and FNAC samples from patients with PTC, indicating its potential as a new diagnostic marker [61]. This finding suggests that miR-30a-5p has the potential to serve as a novel diagnostic marker for PTC, offering an avenue for assessment.

#### miRNAs associated with thyroid cancer classification

Among the miRNAs examined, miR-484, miR-148b-3p, and miR-885-5p have been identified as particularly effective in distinguishing follicular carcinoma [62, 63]. Notably, researchers have observed a significant reduction in miR-15 levels in thyroid cancer cells. Recent studies have suggested a correlation between miR-15 expression and TERT promoter variants in PTCs, which may aid in diagnostic processes [64, 65]. This relationship suggests a potential utility for miR-15 in evaluating the risk of aggressive disease in thyroid cancer patients.

#### Other potential markers

Promising markers in thyroid cancer research are increasingly spotlighting specific miRNAs and their associations with tumor characteristics. One such miRNA, miR-206, demonstrates a negative correlation with CCND2 expression in FNAC samples [11]. Additionally, miR-181b has gained recognition for its potential to differentiate between benign and malignant thyroid nodules [54]. However, the roles of certain target genes of miRNAs, such as miR-148b-3p and miR-484, in the pathogenesis of thyroid cancer still require further investigation.

While the exact role of miR-7 in thyroid cancer remains to be fully elucidated, it has emerged as a valuable supplementary marker, exhibiting a high negative predictive value for differentiating malignant FNAC samples [66]. Its high negative predictive value could reduce unnecessary surgical interventions in cases of benign nodules, thereby improving clinical outcomes. Furthermore, miR-138 has been shown to target TERT, contributing to the differentiation between benign and malignant follicular lesions [67]. Overall, these findings underscore the potential of miRNAs as critical markers.

Our analysis reveals that 11 of the 16 examined miRNAs are up-regulated while five are down-regulated. Approximately two-thirds of these miRNAs exhibit oncogenic properties, with a single miRNA capable of targeting multiple genes, and different miRNAs able to regulate a single gene. Many genes targeted by these miRNAs are involved in intracellular signaling pathways, apoptotic processes, and cell proliferation. Among them, miR-146b emerges as the most promising candidate based on the current literature.

### Long non-coding RNAs

lncRNAs represent a diverse class of RNA molecules characterized by their length exceeding 200 nucleotides and lack of protein-coding capacity. Despite initially being dismissed as transcriptional noise, lncRNAs have emerged as key regulators of gene expression and diverse cellular processes such as regulating DNA methylation [68], control of innate immunity [69], regulating mRNA turnover [70], reprogramming of metabolic energy utilization [71], and angiogenesis [72].

As the importance of lncRNAs in cellular functions becomes clearer, research into their roles across different cancer types, including thyroid cancer, has accelerated. Numerous lncRNAs have demonstrated significant pathological and predictive functions in thyroid cancer [73]. However, many studies have focused exclusively on cell lines and have not been conducted on FNAC samples. Only one study in the literature has examined three lncRNAs in FNAC samples: LRRC52-AS1, LINC02082, and UNC5B-AS1 (Table 3) [73].

#### Specific lncRNAs of interest

LRRC52-AS1 (LRRC52 Antisense RNA 1), belonging to the LRRC superfamily, has been shown to influence the progression of PTC through interactions with mesenchymal markers such as N-cadherin and vimentin. This lncRNA exhibits increased expression in PTC, and reducing LRRC52-AS1 levels can inhibit cellular migration and invasion [74]. In FNAC samples, its expression is also elevated compared to benign nodules [73].

LINC02082 is another important lncRNA linked to the e phosphatidylinositol 3-kinase (PI3K)/Akt signaling pathway through its regulation of *EPHA2*, *LAMB3*, and *LPAR5*. In FNAC samples, LINC02082 expression is markedly higher in PTC compared to benign nodules [73, 75].

Lastly, a study by Wang et al. found that downregulation of UNC5B-AS1 significantly suppresses proliferation, migration, and invasion in PTC cell lines. This lncRNA’s expression levels have also been linked to tumor growth progression and even subtype transformation based on surgical biopsies [76]. In FNAC samples, UNC5B-AS1 is more highly expressed in PTC than in benign nodules [73].

### Current limitations and challenges

Overall, the investigation of ncRNAs, particularly miRNAs and lncRNAs, holds great promise for improving the diagnostic capabilities for thyroid cancer. These molecular markers provide insight into tumor characteristics that go beyond traditional histopathological evaluation, potentially allowing for more personalized treatment approaches. However, there are several technical challenges that must be addressed. The feasibility of implementing these analyses in every clinical center may vary based on available resources, laboratory infrastructure, and expertise in molecular biology techniques.

Currently, while clinicians can use specific molecular markers like miR-146b for thyroid cancer diagnosis, broader integration into routine clinical practice is limited. This limitation stems from uncertainties about the standardization and validation of these markers across larger, diverse patient cohorts. For instance, miR-146b’s efficacy as a marker for PTC can vary significantly based on assay methods, RNA extraction protocols, and patient populations. If a marker is validated in one cohort but not in another, relying on this marker can be problematic.

### Comparison to classical genetic analyses

Compared to classical genetic analyses—such as those that look for well-characterized mutations in genes like *BRAF* or *RAS*—ncRNA profiling can require a more nuanced understanding of gene regulatory networks. Classical genetic tests provide relatively straightforward information (e.g., presence or absence of a mutation) that directly correlates with tumor biology and therapeutic options [77]. In contrast, ncRNA expression can indicate multiple regulatory pathways and target various genes, making it necessary for clinicians to understand not only what miRNAs or lncRNAs are present but also how they interact with each other and influence cellular behavior. For example, while an established mutation in the *BRAF* gene might indicate a higher likelihood of aggressive disease and direct the clinician toward targeted therapy, the implications of elevated levels of several miRNAs or lncRNAs may not be as clear-cut. These markers could contribute to different aspects of tumor behavior, such as proliferation, apoptosis, and metastasis, requiring a more holistic approach to interpretation.

## Conclusion and future directions

Over the past decade, with the advancement of high-throughput molecular biology techniques [78], there has been an increase in the number of molecules exhibiting changes in malignant lesions, leading to a better understanding of thyroid cancer pathogenesis. While the first studies in thyroid FNAC samples started at the protein level through molecules such as galectin-3, over time they evolved from coding RNAs to non-coding RNAs. Particularly, the recognition that non-coding RNAs are not “junk” has led to a shift in the approach to thyroid cancer [79].

The intriguing evidence of dysregulated miRNA and lncRNA in various thyroid neoplasms and their potential utility as sensitive markers for diagnosis and prognosis is noteworthy. By integrating RNA markers into routine clinical workflows, clinicians can achieve earlier diagnoses and tailor treatment plans to individual patient profiles, ultimately improving outcomes. However, the road ahead is still long. The number of samples used in studies is limited, and it is essential to analyze which markers can be used for which subtype of thyroid cancer. While using a single molecule for cancer diagnosis seems ideal, practically, the use of two or more combined molecules yields more meaningful results. Additionally, using a simple method such as real-time PCR appears to be more advantageous. When conducting real-time PCR, it’s essential to consider that variations in the choice of reference genes can influence the reliability of results for both coding and noncoding RNAs. The use of distinct reference genes can lead to substantial discrepancies in the outcomes, underscoring the importance of selecting appropriate and consistent reference genes to ensure accurate and comparable results.

While current research mainly focuses on identifying cancer-specific molecules, future studies should shift towards a patient-specific approach rather than solely a cancer-specific one. Establishing an international database to collect and share data across research centers worldwide would be highly beneficial for validating the sensitivity and specificity of identified markers in diverse patient populations and subtypes of thyroid cancer. However, simply increasing the sample size is not sufficient; it is also important to ensure that the study includes a greater number of samples from the AUS category. Many existing studies have had a limited number of AUS samples or did not include AUS samples at all, relying instead on benign and malignant samples. Additionally, the cost of molecular testing and disparities in healthcare infrastructure limit accessibility, particularly for certain patient populations and resource-limited settings [80].

In our review, we compared several promising RNA markers by grouping them based on their diagnostic performance, as summarized in Table 4. This table offers a comparative overview of mRNAs and miRNAs based on sensitivity, specificity, and overall diagnostic reliability. Notably, the miRNA markers—miR-146b, miR-375, and miR-222—demonstrate distinct performance characteristics. For example, miR-375 exhibits exceptional performance in differentiating MTC, achieving 100% sensitivity and specificity. In contrast, miR-222 has been evaluated in two independent studies utilizing combinations of miRNAs, which underscores the importance of employing multiple markers rather than relying on a single one. Although miR-146b has been recognized as an important marker in various studies, its sensitivity and specificity vary considerably. Among the mRNA markers, *PAPPA* has been reported in only one study and, while promising, requires validation in larger cohorts. Similarly, *TIMP1 and HMGA2* exhibit variable performance, suggesting that their diagnostic value may be maximized when incorporated into a multi-marker panel. This integrated approach, potentially combined with established markers such as the BRAF p.V600E variant, could enhance diagnostic precision and ultimately support a more individualized strategy for patient management.

**Table 4:** Comparative table of promising RNA markers

Marker	Type	Sensitivity (%)	Specificity (%)	Diagnostic Reliability & Clinical Potential
*PAPPA*	mRNA	80	82	Moderate performance in PTC; useful as part of a diagnostic panel
*TIMP1*	mRNA	97.5 (83–100)	90.2 (77–100)	Robust diagnostic performance in thyroid malignancies
*HMGA2*	mRNA	86 (72–100)	93.2 (84.5–100)	Variable performance, diagnostic value appears highest when integrated into a multi-marker panel
miR-146b	miRNA	89.1 (78–100)	87.5 (66–100)	Moderate-to-high diagnostic accuracy
miR-375	miRNA	100	100	Very strong performance in differentiating MTC
miR-222	miRNA	95.5 (90.9–100)	92.3 (86–98.5)	Very strong performance when combined with other miRNAs

*PTC* papillary thyroid cancer, *MTC* medullary thyroid carcinoma, *PAPPA* pregnancy-associated plasma protein A, *TIMP1* tissue inhibitors of metalloproteinase 1, *HMGA2* the high mobility group protein 2

Machine learning offers an additional approach for diagnosing or predicting thyroid nodules. To date, algorithms with satisfactory predictive performance for diagnosing thyroid nodule malignancy have been developed using clinical data and radiological features [81, 82]. A notable advantage of these algorithms is their ability to incorporate diverse diagnostic biomarkers. For instance, Cao et al. demonstrated that factors such as Hashimoto’s thyroiditis, BRAF p.V600E variant status, ill-defined margins, echogenic foci, and ultrasound-based suspicion of cervical lymph node metastasis are critical predictors [83]. Importantly, the BRAF p.V600E variant, as the most extensively studied mutation in thyroid cancer, has been reported to possess an approximate specificity of 100% in PTC [84]. In this context, combining promising markers—such as *PAPPA, TIMP1*, and miR-146b—with BRAF p.V600E and clinical parameters may be a viable strategy for developing superior diagnostic algorithms.

It is necessary to emphasize that while the Bethesda III/IV nodules have become a brighter ‘moon’ than before, it still has its ‘dark side’. Continued research, enhanced diagnostic tools, and a multidisciplinary approach are essential to illuminate these uncertainties and improve patient management.
